# Blood transcriptome analysis revealed the crosstalk between COVID-19 and HIV

**DOI:** 10.3389/fimmu.2022.1008653

**Published:** 2022-10-28

**Authors:** Cheng Yan, Yandie Niu, Xuannian Wang

**Affiliations:** School of Pharmacy, Key Laboratory of Nano-carbon Modified Film Technology of Henan Province, Diagnostic Laboratory of Animal Diseases, Xinxiang University, Xinxiang, Henan, China

**Keywords:** COVID-19, HIV, common differentially expressed genes, hub genes, small molecular compounds

## Abstract

**Background:**

The severe coronavirus disease 2019 (COVID-19) is an infectious disease caused by the severe acute respiratory syndrome coronavirus 2 (SARS-CoV-2), which has resulted in the most devastating pandemic in modern history. Human immunodeficiency virus (HIV) destroys immune system cells and weakens the body’s ability to resist daily infections and diseases. Furthermore, HIV-infected individuals had double COVID-19 mortality risk and experienced worse COVID-related outcomes. However, the existing research still lacks the understanding of the molecular mechanism underlying crosstalk between COVID-19 and HIV. The aim of our work was to illustrate blood transcriptome crosstalk between COVID-19 and HIV and to provide potential drugs that might be useful for the treatment of HIV-infected COVID-19 patients.

**Methods:**

COVID-19 datasets (GSE171110 and GSE152418) were downloaded from Gene Expression Omnibus (GEO) database, including 54 whole-blood samples and 33 peripheral blood mononuclear cells samples, respectively. HIV dataset (GSE37250) was also obtained from GEO database, containing 537 whole-blood samples. Next, the “Deseq2” package was used to identify differentially expressed genes (DEGs) between COVID-19 datasets (GSE171110 and GSE152418) and the “limma” package was utilized to identify DEGs between HIV dataset (GSE37250). By intersecting these two DEG sets, we generated common DEGs for further analysis, containing Kyoto Encyclopedia of Genes and Genomes (KEGG) pathway and Gene Ontology (GO) functional enrichment analysis, protein-protein interaction (PPI) analysis, transcription factor (TF) candidate identification, microRNAs (miRNAs) candidate identification and drug candidate identification.

**Results:**

In this study, a total of 3213 DEGs were identified from the merged COVID-19 dataset (GSE171110 and GSE152418), and 1718 DEGs were obtained from GSE37250 dataset. Then, we identified 394 common DEGs from the intersection of the DEGs in COVID-19 and HIV datasets. GO and KEGG enrichment analysis indicated that common DEGs were mainly gathered in chromosome-related and cell cycle-related signal pathways. Top ten hub genes (CCNA2, CCNB1, CDC20, TOP2A, AURKB, PLK1, BUB1B, KIF11, DLGAP5, RRM2) were ranked according to their scores, which were screened out using degree algorithm on the basis of common DEGs. Moreover, top ten drug candidates (LUCANTHONE, Dasatinib, etoposide, Enterolactone, troglitazone, testosterone, estradiol, calcitriol, resveratrol, tetradioxin) ranked by their P values were screened out, which maybe be beneficial for the treatment of HIV-infected COVID-19 patients.

**Conclusion:**

In this study, we provide potential molecular targets, signaling pathways, small molecular compounds, and promising biomarkers that contribute to worse COVID-19 prognosis in patients with HIV, which might contribute to precise diagnosis and treatment for HIV-infected COVID-19 patients.

## Introduction

The COVID-19 virus caused by the SARS-CoV-2 virus emerged in Wuhan, China, in December 2019, resulting in a large loss of human life all over the world and posing a serious threat to public health, food systems and the world of work (1–4). A recent World Health Organization (WHO) report conducted in China showed fever, diarrhea, sore throat, dry cough, and fatigue were listed as the most common COVID-19 symptoms, while musculoskeletal symptoms, including arthralgia and muscular aches may also occur by infecting coronavirus (5–9). It is common for patients suffering from severe COVID-19 cases to suffer from acute respiratory distress syndrome (ARDS) and respiratory failure, organ manifestations that lead to the majority of fatalities resulting from COVID-19 (10). Due to COVID’s rapidly evolving mutant strains, the difficulties of manufacturing vaccines, and the lack of vaccines, the fight against COVID-19 remains highly challenging (11).

Acquired immunodeficiency syndrome (AIDS) is defined as less than 200 CD4 T-cells per liter of blood or as a disease that defines AIDS (12, 13). Infection with HIV causes human immunodeficiency virus infection and acquired immunodeficiency syndrome (HIV/AIDS) (14). The World Health Organization estimates that as of 2021, HIV/AIDS has killed about 36.3 million people and affected approximately 37.7 million people worldwide (15).

Previous studies showed that HIV-infected individuals had double COVID-19 mortality risk and experienced worse COVID-related outcomes (16–18). According to a recent study, HIV was also an independent risk factor for both severe/critical COVID-19 at admission and in-hospital mortality (19). Therefore, it is essential to explore potential molecular mechanisms and screen out potential small molecular compounds for the treatment of HIV-infected COVID-19 patients.

In addition, white blood cells circulating around the blood help the immune system fight off infections and act as a first line defense against disease-causing microorganisms. HIV infection is characterized by CD4+ T cell depletion, CD8+ T cell expansion, and chronic immune activation that leads to immune dysfunction (20). COVID-19 can trigger a cytokine storm in pulmonary tissues through hyperactivation of the immune system and the uncontrolled release of cytokines (21). These results indicate that blood cells may play important role in the progression of COVID-19 in HIV-infected individuals. The purpose of our work was to illustrate blood transcriptome crosstalk between COVID-19 and HIV and to provide potential drugs that might be useful for the treatment of HIV-infected COVID-19 patients.

Herein, we identified 394 common DEGs from the intersection of the DEGs in COVID-19 and HIV datasets. Next, the common DEGs were analyzed using KEGG and GO functional enrichment analysis to identify potential biological pathways. Then, PPI network was developed to identify hub genes that may serve as diagnostic markers for disease. Subsequently, for the advancement of COVID-19 and HIV clinical diagnosis and treatment, we analyzed TFs, miRNAs on the basis of the common DEGs. Finally, we explored possible small molecule compounds that may be profitable for treating HIV-infected COVID-19 patients.

## Methods

### Data acquisition

One COVID-19 dataset (GEO accession ID: GSE171110) consisted of 44 COVID-19-infected whole-blood samples and 10 healthy whole-blood samples with Illumina HiSeq 2000 (22). Another COVID-19 dataset (GEO accession ID: GSE152418) contained 16 peripheral blood mononuclear cells samples and 17 healthy samples (23). Similarly, the HIV dataset (GEO accession ID: GSE37250) consisted of 274 positive HIV-infected whole-blood samples, 263 negative HIV-infected whole-blood samples.

### Identification of common DEGs

The “Deseq2” package in RStudio software (version 4.1.2) was performed to select DEGs with |Log_2_ Fold Change| > 0.585 and | adj.P.Val. | < 0.05 for COVID-19 datasets (GSE171110 and GSE152418). Meanwhile, the “limma” package was used to identify DEGs with |Log_2_ Fold Change| ≥ 0.1 and | adj.P.Val. | <0.001 for HIV dataset (GSE37250). Using “venn” package in R software, we obtained COVID-19 and HIV common DEGs.

### Functional enrichment analysis of common DEGs

The “clusterProfiler” package in RStudio software (version 4.1.2) was performed to explore the possible biological pathways of the common DEGs with GO and KEGG enrichment analysis. P-value < 0.05 was used for quantifying the top listed functional items and pathways of common DEGs.

### Analysis of PPI network based on common DEGs

The interactions among proteins are represented by PPI networks, which are crucial to understanding cell physiology in health and disease. It is essential to understand and gain insight into the cellular machine process by examining PPI network function and its interaction with cellular machinery (24). A set of common DEGs was also uploaded to the STRING website (https://string-db.org/) so that the interactions among proteins could be assessed critically. The PPI network of these common DEGs was constructed based on a combined score larger than 0.9. Then, the PPI networks were visualized using Cytoscape (version 3.9.1) software.

### Extraction of hub genes

Using Cytoscape’s plug-in CytoHubba, we identified the top 10 hub genes by ranking them (25). Using network metrics, Cytoscape allows users to evaluate and identify biological network modulators (26). Furthermore, receiver operating characteristic curve (ROC) analyses based on degree algorithms were conducted on the top 10 hub genes.

### Recognition of transcription factors and MiRNAs

Proteins called TFs can bind to specific DNA sequences and form a complex regulatory system that controls genome expression (27). Enrichr is a web-based enrichment analysis tool that provides different types of visual summary summaries for gene lists (28). We used the Enrichr Transcription Factor PPIs library to identify TFs in common DEGs and developed a TF-gene interaction map using Cytoscape software (version 3.9.1). In addition, target gene-miRNA interaction analysis was performed to detect miRNAs that could successfully attach to target gene transcripts and negatively have an influence on the expression of protein through destabilizing mature messenger RNA and reducing corresponding translation efficiency (29). MiRTarBase provides information about miRNA-target interactions (MTIs) that have been experimentally validated (30). Then, we used the Enrichr miRTarBase 2017 library to identify miRNAs in common DEGs and developed a miRNA-gene interaction map using Cytoscape software (version 3.9.1).

### Evaluation of applicant drugs

An online resource, Drug Signatures Database (DSigDB), connects drugs/compounds to their target genes (31). To study the drug molecular properties of COVID-19 and HIV, we used the Drug Signatures Database (DSigDB) library under the Diseases/Drugs function in Enrichr (https://maayanlab.cloud/Enrichr/enrich).

### Gene-disease association analysis

The DisGeNET database contains one of the most comprehensive collections of genes and variants associated with human disease (32–35). Based on common DEGs, we identified diseases and chronic health problems using DisGeNET database under the Diseases/Drugs function in Enrichr.

## Results

### Identification of DEGs and common DEGs between COVID-19 and HIV

#### Datasets

**Figure 1:**
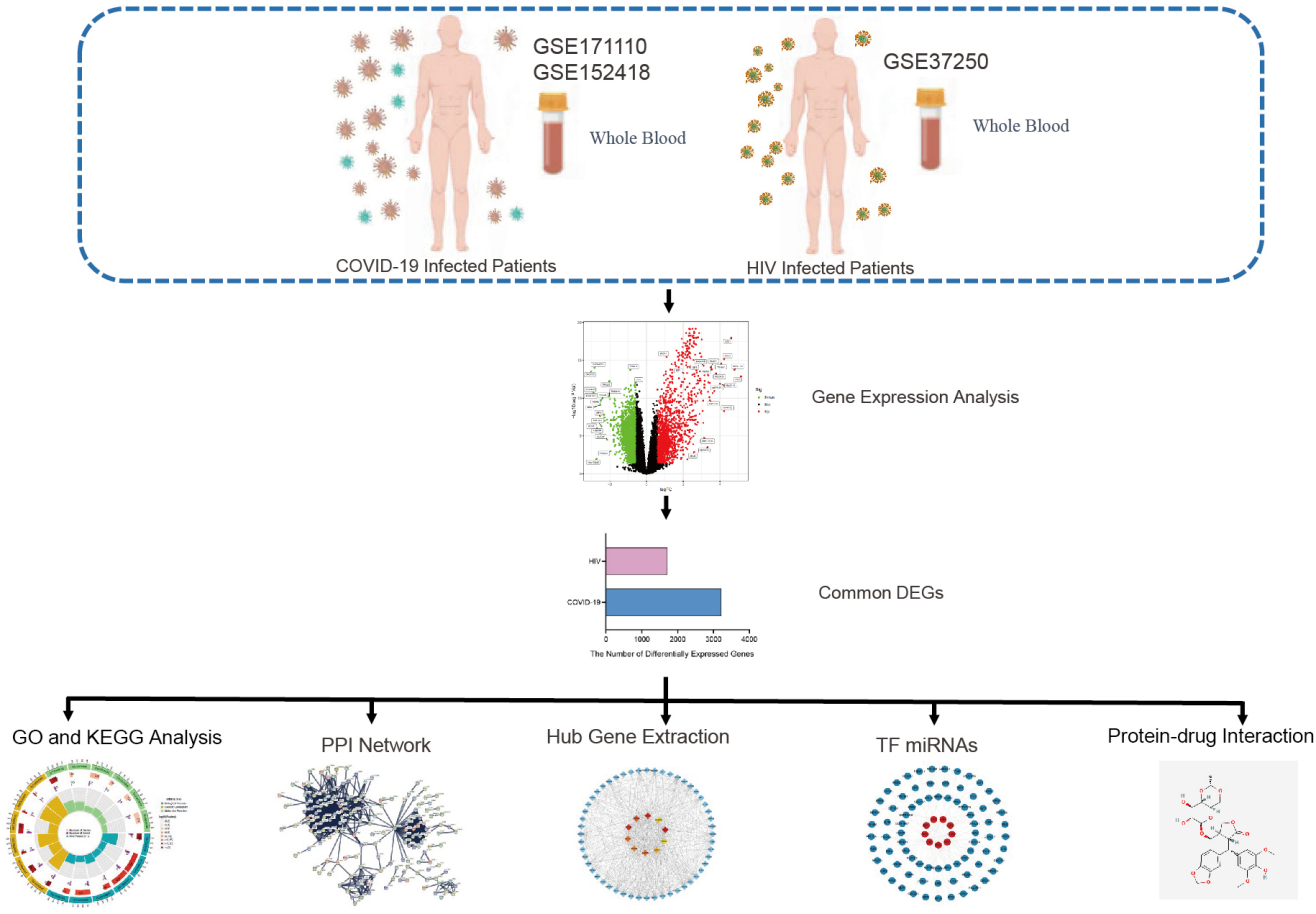
The entire workflow diagram of our research.

**Figure 2:**
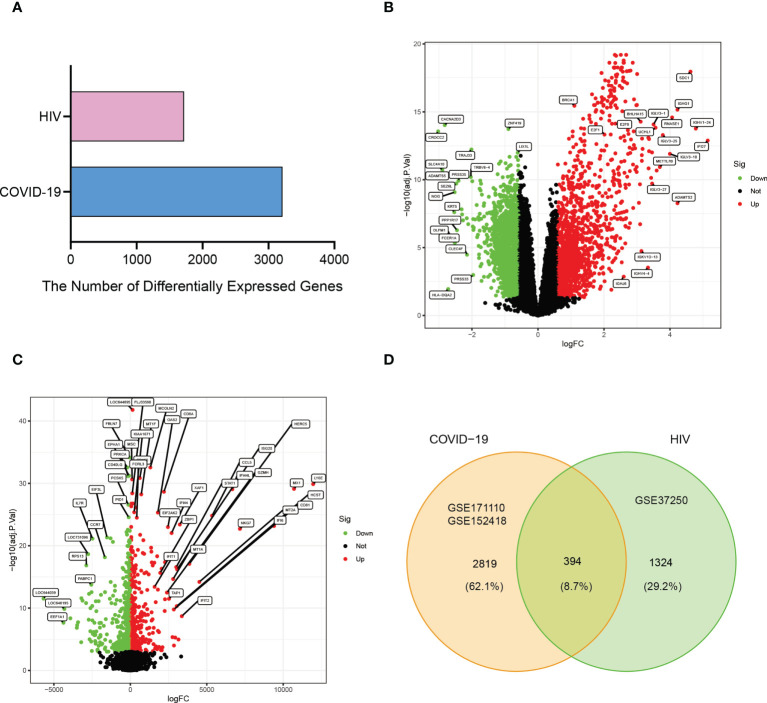
Visualization the number of common differentially expressed genes between two datasets, COVID-19 (GSE171110 and GSE152418) and HIV (GSE37250). **(A)** Comparing the number of differentially expressed genes between COVID-19 and HIV. **(B)** The volcano plot of differentially expressed genes in COVID-19 datasets. **(C)** The volcano plot of differentially expressed genes in the HIV dataset. **(D)** Venn diagram showing the overlap of differentially expressed genes between COVID-19 and HIV.

A flowchart was created to depict all the critical and significant processes of our study (**Figure 1**). In order to investigate the interactions between COVID-19 and HIV, we analyzed the blood samples downloaded from GEO database. Box plots of the gene expression of COVID-19 and HIV datasets before and after normalization were shown in **Supplementary Figures 4**-**6**. In the COVID-19 dataset, we identified 3213 DEGs with|Log_2_ Fold Change| > 0.585 and | adj.P.Val. | < 0.05, whereas in the HIV dataset, we identified 1718 DEGs with |Log_2_ Fold Change| ≥ 0.1 and | adj.P.Val. | <0.001 (**Figure 2A**; **Supplementary Tables 1**, **2**). Moreover, the volcano plots depicted DEGs of COVID-19 and HIV, respectively (**Figures 2B, C**; **Supplementary Tables 3**, **4**). Venn diagram revealed that 394 DEGs were common in both COVID-19 and HIV datasets (**Figure 2D**). According to these findings, HIV and COVID-19 have a large number of common genes and are closely related.

### Functional enrichment analysis of common DEGs

**Figure 3:**
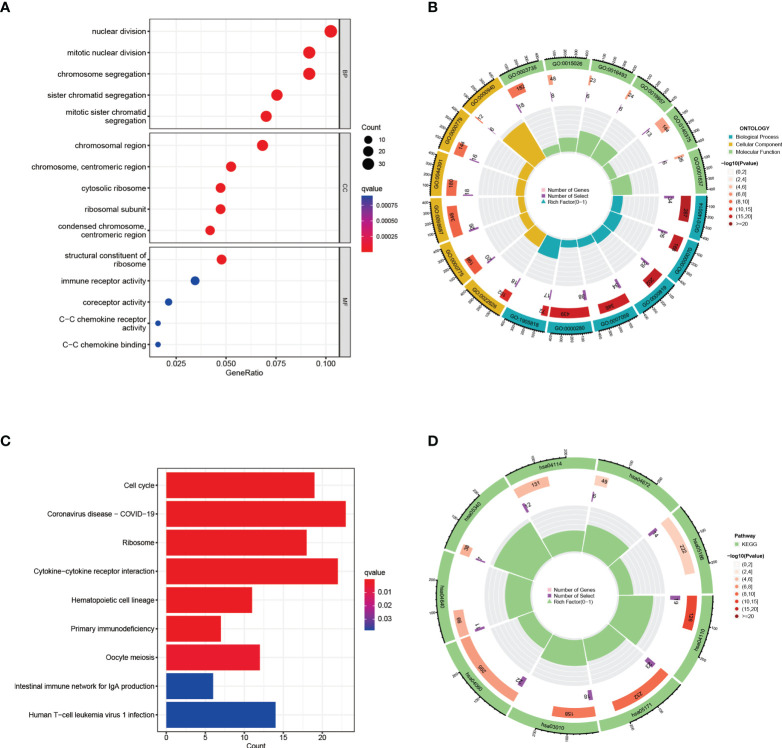
GO and KEGG functional enrichment analysis of the common differentially expressed genes between COVID-19 and HIV. **(A)** The bubble graphs of GO enrichment analysis. **(B)** Circle diagram of GO enrichment analysis. **(C)** The bar graphs of KEGG enrichment analysis. **(D)** Circle diagram of KEGG enrichment analysis.

The GO enrichment analyses are commonly used to show the interactions between genes and terms, whereas KEGG enrichment analyses can illustrate the relationship between genes and patterns of function (36). “ClusterProfiler” package was used to discover biological features and pathways that were enriched in this work as common DEGs. GO enrichment analysis showed significantly enriched pathways (p-value < 0.05), biological process (BP), cell composition (CC), and molecular function (MF) are included. Notable pathways among BP category were mitotic nuclear division, mitotic sister chromatid segregation, and sister chromatid segregation. In the CC category, the top three terms were cytosolic ribosome, chromosome, centromeric region and chromosomal region. Moreover, in the MF aspect, structural constituent of ribosome, coreceptor activity, and C−C chemokine receptor activity were the top three statistically significant GO terms (**Figures 3A, B** **)**. The top three pathways of KEGG enrichment analysis were Coronavirus disease-COVID-19, Cytokine−cytokine receptor interaction, and Cell cycle (**Figures 3C, D** **)**. These results suggest that these common DEGs have strong relationships with cell cycle, which may lead to a more effective treatment for COVID-19 and HIV.

### PPI network and hub genes extraction of common DEGs

**Figure 4:**
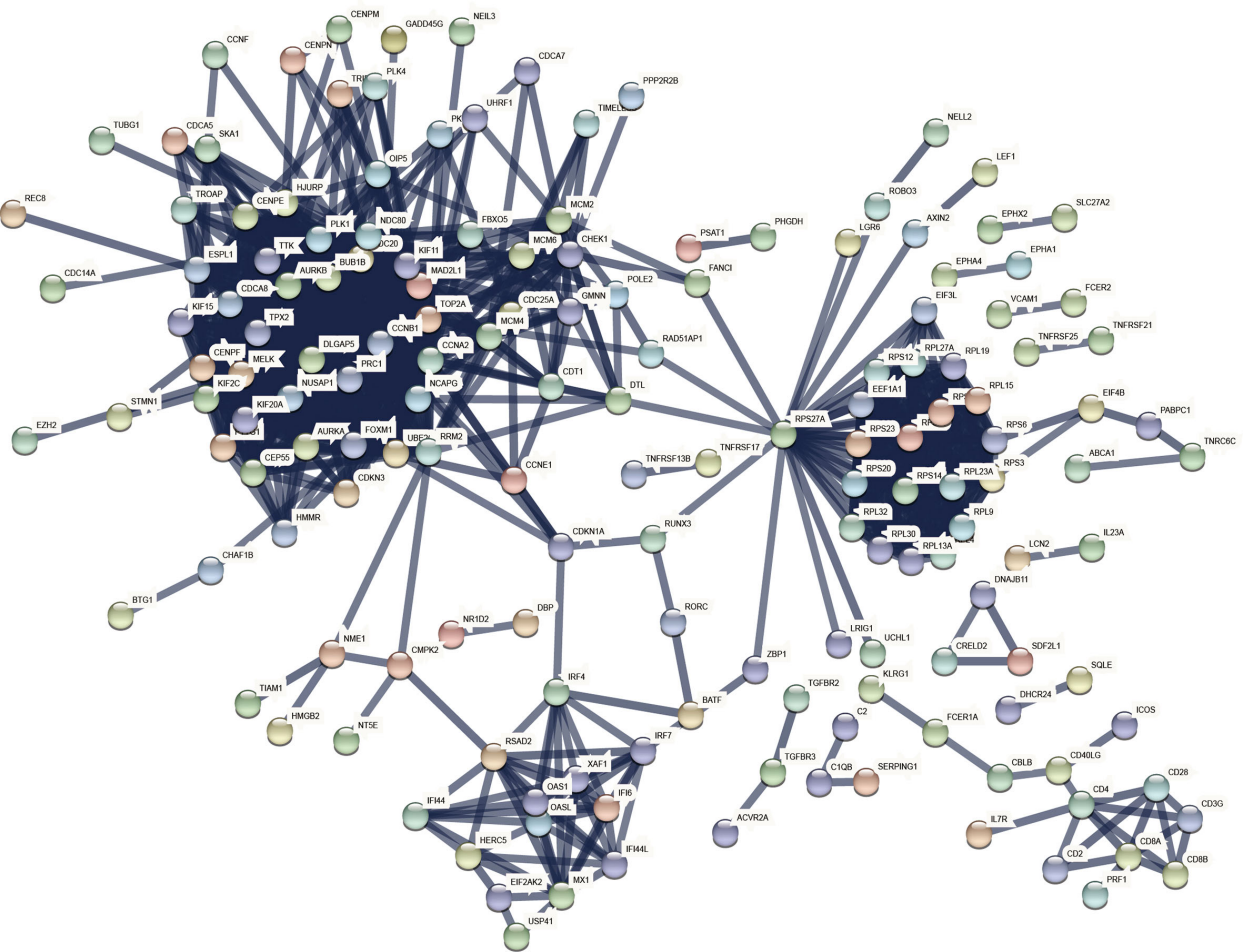
COVID-19 and HIV common differentially expressed genes in the PPI network.

**Figure 5:**
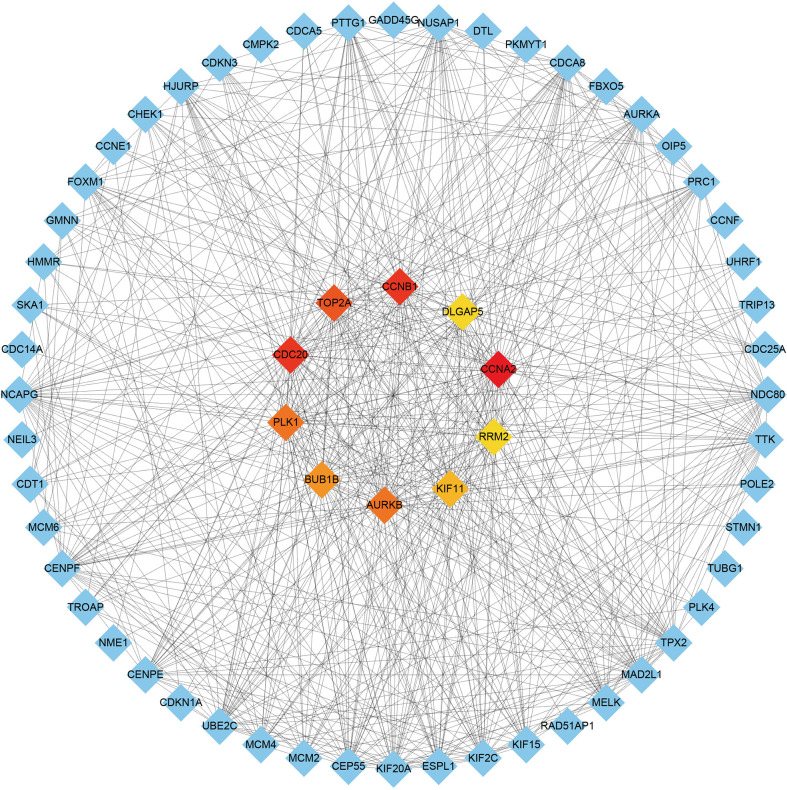
Identification of the top 10 hub genes ranked by their scores. In the middle circle, the 10 central genes represent the top 10 hub genes ranked by their scores and their interactions with other common DEGs.

A comprehensive analysis of these common DEGs between COVID-19 and HIV was conducted using STRING to explore protein-protein interactions. **Figure 4** illustrates the interactions between the common DEGs in HIV and COVID-19. As shown in **Figure 5**, top 10 hub genes were ranked according to their scores, which were identified using degree method based on the results of PPI network in Cytoscape, including CCNA2, CCNB1, CDC20, TOP2A, AURKB, PLK1, BUB1B, KIF11, DLGAP5, RRM2. Next, ROC analysis was conducted for HIV and COVID-19, respectively. Area under curve (AUC) values for all hub genes in the HIV dataset were greater than 0.616, whereas AUC values for all hub genes in the COVID-19 dataset were greater than 0.973 (**Supplementary Figures 2**, **3**). According to these results, it is potential to develop novel targeted therapies against COVID-19 by targeting these hub genes.

### Construction of regulatory networks

**Figure 6:**
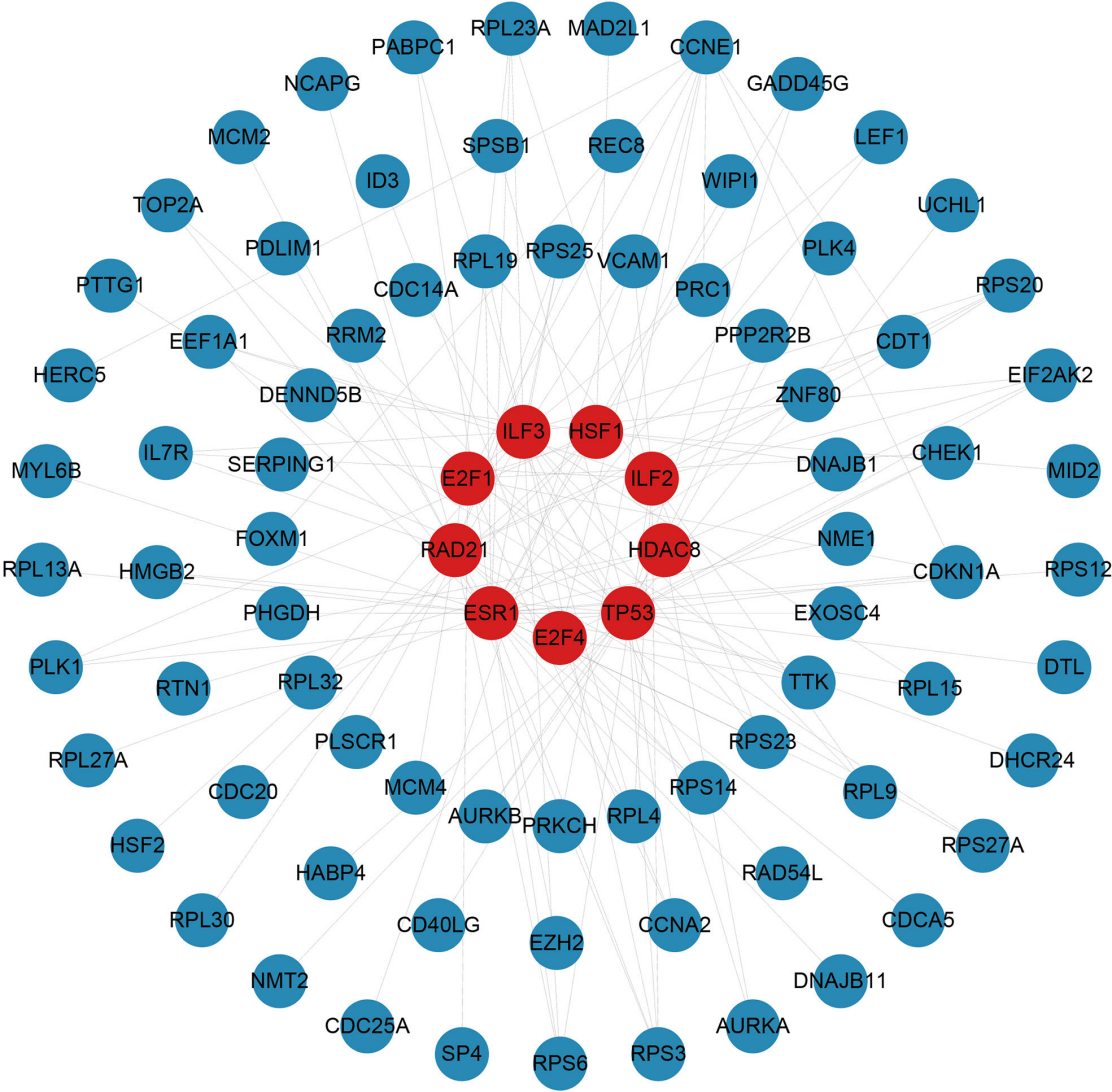
The top 10 transcription factors ranked according to their P values and their interactions with common differentially expressed genes. In this network, the red circles represent transcription factors with the top 10 lowest P values in the network, while the blue circles represent common differentially expressed genes correlated with transcription factors.

**Figure 7:**
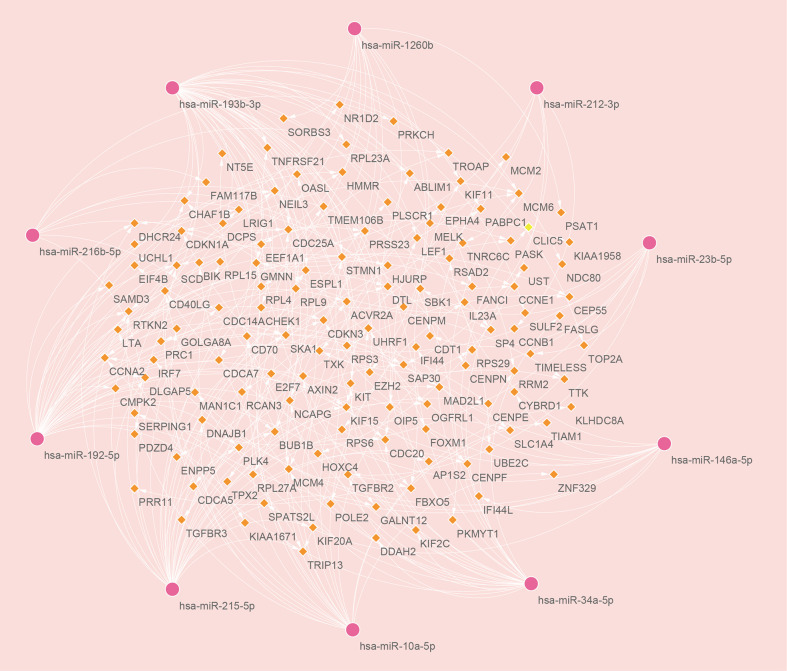
The top ten microRNAs with the top 10 lowest P values and their interactions with common differentially expressed genes. In this network, the pink circles represent miRNAs with the top 10 lowest P values, while the orange diamonds represent common differentially expressed genes correlated with miRNAs.

There are two types of gene expression regulators: transcription factors (TFs) and miRNAs. TFs modulate transcription by binding the promoter regions, whereas miRNAs regulate post-transcriptional gene expression (37). Analysis of interactions between TFs and miRNAs revealed that 165 TFs and 2466 miRNAs coordinate these common DEGs, indicating that they cooperate heavily. As shown in **Figure 6**, the top ten TFs were ranked according to their P values, including ILF3, RAD21, ILF2, TP53, CCNE1, E2F4, E2F1, HDAC8, ESR1, and HSF1. The top ten miRNAs were also ranked by their P values, containing hsa-miR-193b-3p, hsa-miR-192-5p, hsa-miR-215-5p, hsa-miR-146a-5p, hsa-miR-10a-5p, hsa-miR-216b-5p, hsa-miR-212-3p, hsa-miR-34a-5p, hsa-miR-1260b, and hsa-miR-23b-5p (**Figure 7**). These findings indicated that there was strong relationship between common DEGs and TFs, miRNAs.

### Identification of candidate drugs

**Figure 8:**
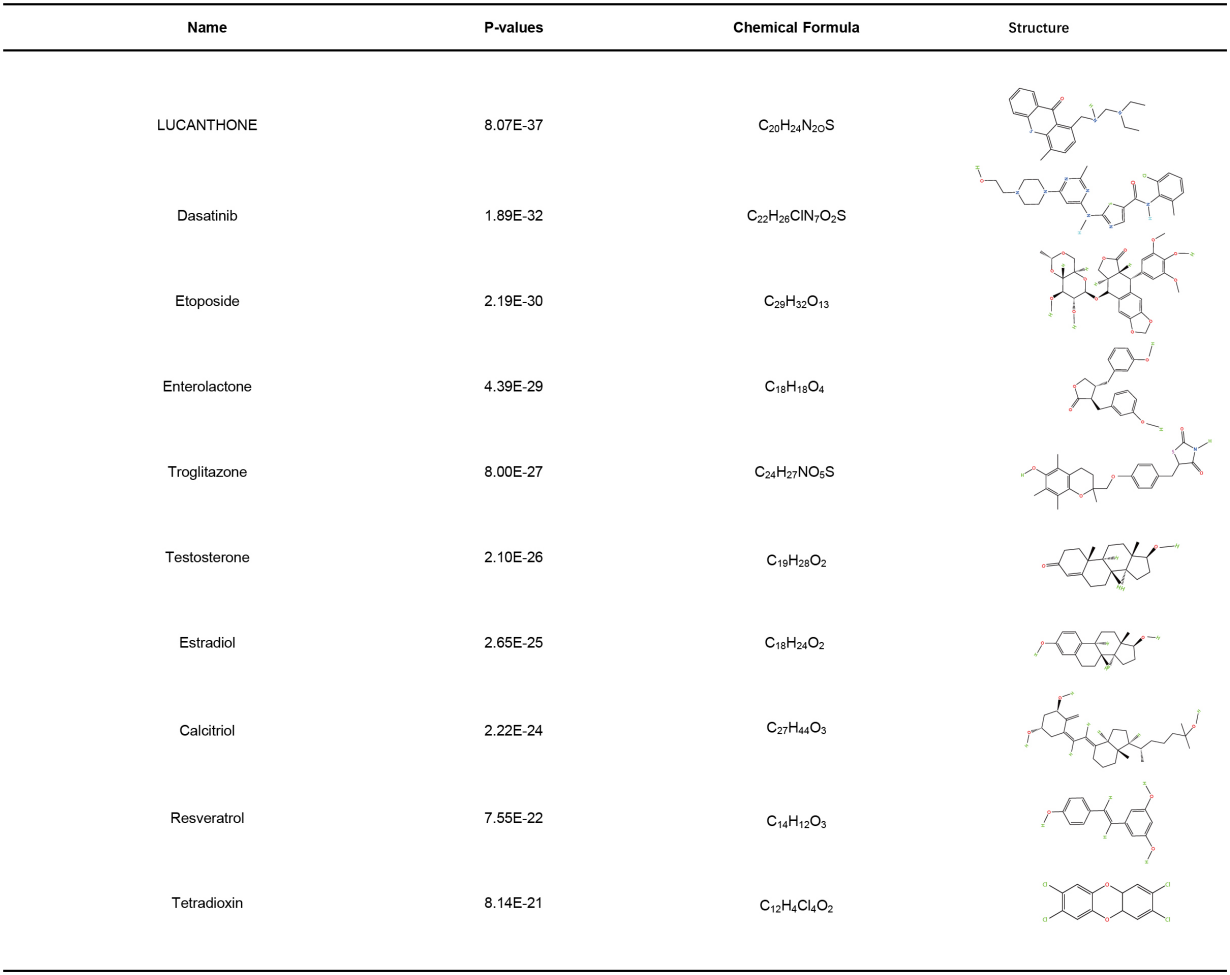
Identification of potential drugs.

As shown in **Figure 8**, top ten drugs were ranked according to their P values (LUCANTHONE, Dasatinib, etoposide, Enterolactone, troglitazone, testosterone, estradiol, calcitriol, resveratrol, tetradioxin), which were identified from the DSigDB library in Enrichr based on P-value. These potential small molecular compounds might serve as therapeutic targets and treatment for COVID-19 and HIV.

### Identification of disease association

It has been shown that various diseases are linked and must have at least one or more genes in common (38). Based on DisGeNET library in Enrichr, we screened out five diseases that has strong relationship with common DEGs, including malignant neoplasm of breast, breast carcinoma, malignant lymphoma, lymphocytic, intermediate differentiation, diffuse, nasopharyngeal carcinoma, and leukemia (**Supplementary Figure 1**). These results indicated these diseases have something in common with HIV and COVID-19.

## Discussion

A growing number of studies have demonstrated possible connections between various diseases in recent years. Interactions between different diseases are therefore a highly promising field that needs to be investigated in the future (23, 39, 40). There have been dramatic deaths worldwide due to COVID-19, which has presented a major challenge to public health, the food system, and the workforce. HIV is a member of the family Retrovirae within the genus Lentivirus (41), which has a wide-ranging impact on individuals’ health, households, communities, and nations’ economic and social well-being (42). People living with HIV have badly compromised immune system. The recent studies showed that patients living with HIV had a higher risk of SARS-CoV-2 infection and mortality risk from COVID-19 than people without HIV (19, 43–45). HIV was identified as an independent risk factor for both severe-type and critical-type COVID-19 (46). However, the molecular mechanisms that contribute to worse COVID-19 prognosis in patients with HIV are still unclear. In this study, we downloaded COVID-19 datasets (GSE171110 and GSE152418) and HIV dataset (GSE37250) from GEO database. Next, we performed differential analysis on COVID-19 and HIV datasets, respectively. Then, by intersecting these two DEG sets, we generated common DEGs for further analysis, and identified 10 hub genes ranked according to their scores (CCNA2, CCNB1, CDC20, TOP2A, AURKB, PLK1, BUB1B, KIF11, DLGAP5, and RRM2) using degree method. Hub proteins could be used for the development of therapeutic intervention. Cyclin A2 (CCNA2) regulates the cell cycle and contributes to tumor growth (47). Cyclin B1 (CCNB1) is a crucial mitosis initiator and controller (48). CDC20 is a well-known regulator of cell division, regulating chromosome segregation during mitosis (49). The mitotic chromosome condensation is established by topoisomerase IIα (TOP2A), a core component of mitotic chromosomes (50). Aurora kinase B (AURKB) has been identified in Caco-2 cells as a DEG of SARS-CoV-2 (51). The Polo-like Kinase 1 (PLK1) is an essential enzyme in mitosis, which initiates, maintains, and completes it (52). Moreover, knockdown of BUB1B caused acute chromosomal abnormalities as well as impaired chromosome alignment (53). A kinesin called KIF11 is responsible for intracellular vesicle transport and mitosis, as well as being overexpressed in tumors (54). DLGAP5, a gene mapped to chromosome 14q22.3, plays an important role in cancer formation (55). During DNA replication, RRM2 is essential for synthesis of Deoxynucleoside triphosphate (dNTP) in S phase of cell cycle (56).

As previous studies indicated that lower CD4 count in people living with HIV strongly correlated with increased odds of SARS-CoV-2 positivity (46). These data highlight the urgent need for mechanism studies to better illustrate how HIV-associated immunocompromise influences infection acquisition and clearance. In this study, we conducted GO and KEGG analyses to examine the association between COVID-19 and HIV. GO analysis was performed using the “clusterProfiler” package for three types of BP, CC, and MF. The BPs of these common DEGs were primarily enriched in mitotic nuclear division, mitotic sister chromatid segregation, and sister chromatid segregation. The process of chromosome segregation is crucial to the cell cycle (57). SARS-CoV-2 infection may modulate several proteins involved in inflammatory responses and chromosomal segregation (51). The CCs of these common DEGs were mainly enriched in cytosolic ribosome, chromosome, centromeric region and chromosomal region. The MF results indicated that these common DEGs were primarily enriched in structural constituent of ribosome, coreceptor activity, and C−C chemokine receptor activity. Cancer, developmental disorders, and virus infections all affect ribosome production, which is universally important in biology (58). Virus relies solely on polypeptide synthesis by ribosomes to produce polypeptide molecules (59, 60). Coronavirus disease-COVID-19, Cytokine−cytokine receptor interaction, and Cell cycle were the top 3 KEGG pathways. In a cell cycle, a specific sequence of events takes place such as cell division, DNA replication, nuclear membrane rupture, spindle formation, and preparation for chromosome segregation (61). A large number of studies have shown that viral replication and survival are facilitated by viruses regulating host cell cycle processes (62–65). Identifying significant gene ontology and molecular pathways improved our understanding of the mechanism for how HIV infection is a risk factor for increased mortality from COVID-19.

We also explored the relationships between TFs, miRNAs and common DEGs. MiRNAs, which are small non-coding RNAs with 20-24 nucleotides, are known to suppress the expression of target genes *via* binding to complementary target sites in the 3’ untranslated region of mRNA post-transcriptionally (37). In many biological processes, miRNAs regulate target genes, and some of these genes may promote cancer or suppress tumor growth (66). A TF is a protein that binds specific DNA sequences to regulate transcription and gene expression (67). In many biological processes, TFs play a key role by binding specific sequences of genes, for example, regulating gene transcription, controlling metabolism, and influencing immunity (68). The top 10 TFs ranked by their P values were ILF3, RAD21, ILF2, TP53, CCNE1, E2F4, E2F1, HDAC8, ESR1, and HSF1. The top ten miRNAs ranked according to their P values were hsa-miR-193b-3p, hsa-miR-192-5p, hsa-miR-215-5p, hsa-miR-146a-5p, hsa-miR-10a-5p, hsa-miR-216b-5p, hsa-miR-212-3p, hsa-miR-34a-5p, hsa-miR-1260b, and hsa-miR-23b-5p. HIV infection is promoted by ILF3 and ILF2 *via* direct interaction with the vRNA (69). The ILF3 suppresses HIV-1 innate sensing as well as other PAMPs that stimulate TLR7/8 and cGAS (70). Inhibition of HDAC does not enhance HIV spread ability, since it does not increase HIV infection susceptibility in peripheral blood mononuclear cells (71). TP53 regulates viral replication and induces G2 cell cycle arrest by interacting with HIV-1 viral infectivity factor (72). The estrogen receptor-1 (ESR-1) regulates HIV-1 latency based on the results of unbias shRNA library screening (73). HSF1 plays an important role in HIV transcription and HIV latent reactivation (74). Among all the miRNAs, SARS-COV infection has been linked to hsa-miR-193b-3p (75) in studies, the effect of other miRNAs for COVID-19 and HIV need to be further explored.

Moreover, we conducted gene-disease analyses to identify common DEGs associated with diseases. According to the results, common DEGs are associated with various types of diseases in HIV and COVID-19, including malignant neoplasm of breast, breast carcinoma, malignant lymphoma, lymphocytic, intermediate differentiation, diffuse, nasopharyngeal carcinoma, and leukemia. It has been recognized for more than 100 years that aneuploidy is a hallmark of tumorigenesis (76). Human cancer is characterized by chromosomal instability, which is associated with poor prognosis, metastasis, and therapeutic resistance (77). Studies showed COVID-19 was highly related to a variety of neoplasms, including mammary, colonic, stomach, and prostatic neoplasms (25). This was consistent with our above results. As a result of the severe nature of cancer and compromised immunity, COVID-19 patients were at a higher risk of dying (78).

Although molnupiravir, paxlovid, and remdesivir are approved by FDA for the treatment of COVID-19, at this time there is no evidence to suggest that any particular antiretroviral therapy agent improves or worsens COVID-19 clinical outcomes in PLHIV, or can be used for prevention of SARS-CoV-2 infection (79). Developing a safe and effective drug for the treatment of HIV-infected COVID-19 patients still has a high priority. In this study, we identified a variety of chemical compounds and drugs that could potentially treat COVID-19 and HIV, including LUCANTHONE, Dasatinib, etoposide, Enterolactone, troglitazone, testosterone, estradiol, calcitriol, resveratrol, tetradioxin. A TOP II inhibitor, Etoposide, prevents intracellular replication of the structural proteins of SARS-CoV-2 while rescuing COVID-19’s cytokine storm (80, 81). There was an early, nondurable clinical benefit associated with immediate etoposide treatment in adults with Kaposi sarcoma and HIV infection (82). The kinase signaling pathway inhibitor Dasatinib is effective against SARS-CoV (83), HIV replication could also be suppressed by Dasatinib by blocking reverse transcription and integration (84, 85). It has been found that troglitazone inhibits SARS-CoV-2 NSP9, which is crucial for viral replication (86). According to increasing evidence, Estradiol participates in the regulation of HIV infection (87), and shows a significant improvement regarding fatality in COVID-19 (88). A recent study found that resveratrol significantly reduced HIV-1 infection in CD4 T cells by cutting off the production of reverse transcripts (89). The use of calcitriol as a treatment for COVID-19 has recently been proposed (90). It has been shown that testosterone reduces COVID-19 levels by inhibiting proinflammatory cytokines, increasing antiinflammatory cytokines, modulating immune system and attenuating oxidative stress and endothelial dysfunction *via* its action on inflammatory cytokines (91). A deficiency of testosterone might contribute to HIV-associated lipodystrophy, or be involved in its pathogenesis (92).

Actually, blood cells are a diverse group of immune cells that act as a first line defense against infections and disease-causing microorganisms. Commonly, People living with HIV who have a CD4+ cell count below 200 are at high risk of developing serious illnesses (93). Consistently, a significant decrease in CD3+ T cells, CD4+ T cells, CD8+ T cells and natural killer cells in PBMCs indicates the severity of COVID-19 patients compared to moderate patients (94). To the best of our knowledge, the molecular blood biomarkers for HIV-infected COVID-19 patients are not reported yet. In this study, the top ten hub genes ranked according to their scores (CCNA2, CCNB1, CDC20, TOP2A, AURKB, PLK1, BUB1B, KIF11, DLGAP5, and RRM2) were analyzed by ROC analysis. All these hub genes in the cohort HIV have an AUC value above 0.616 as a result of our analysis, while in the cohort COVID-19 these hub genes had AUC values above 0.973. The discovery of these molecular blood biomarkers may provide new insights into HIV-infected COVID-19 patients’ diagnosis, care, and treatment.

In summary, our study had many advantages. Firstly, we utilized blood samples of COVID-19 and HIV from GEO database to identify hub genes, which may play crucial roles in the occurrence and development of COVID-19 and HIV. Secondly, we clarified the interactions between CVOID-19 and HIV, which could provide novel insights into the molecular mechanism underlying viral infection with COVID-19 and HIV. Thirdly, we screened out ten candidate drugs ranked by their P values, which might serve as biomarkers for the treatment of COVID-19 and HIV patients.

However, despite the strength of our study, there are several limitations. Firstly, we just derived the research data from the GEO public database. Secondly, the biological functions of hub genes as well as the effectiveness and safety of candidate drugs should be confirmed through basic experiments or clinical trials. Thirdly, the molecular mechanism of COVID-19 and HIV are required to be further investigate.

## Conclusion

In this study, we provide potential molecular targets, signaling pathways, small molecular compounds, and promising biomarkers that contribute to worse COVID-19 prognosis in patients with HIV, which might contribute to precise diagnosis and treatment for HIV-infected COVID-19 patients.

## Data availability statement

The original contributions presented in the study are included in the article/**Supplementary Material**. Further inquiries can be directed to the corresponding authors.

## Author contributions

CY and XW contributed to the conception of the study and data analysis. YN performed the data analyses and wrote the manuscript. All authors contributed to the article and approved the submitted version.

## Funding

This work was supported by the Natural Science Foundation for Young Scientists of Henan Province, China (Grant No. 222300420261).

## Acknowledgments

The authors thank participants and staff of Xinxiang University for their contributions.

## Conflict of interest

The authors declare that the research was conducted in the absence of any commercial or financial relationships that could be construed as a potential conflict of interest.

## Publisher’s note

All claims expressed in this article are solely those of the authors and do not necessarily represent those of their affiliated organizations, or those of the publisher, the editors and the reviewers. Any product that may be evaluated in this article, or claim that may be made by its manufacturer, is not guaranteed or endorsed by the publisher.

## Supplementary material

Supplementary Figure 1 Regulatory interactions between common DEGs and diseases.

Supplementary Figure 2 ROC analysis of the hub DEGs in the HIV dataset.

Supplementary Figure 3 ROC analysis of the hub DEGs in the COVID-19 dataset.

Supplementary Figure 4 Box plots of the gene expression of GSE37250 dataset before **(A)** and after **(B)** normalization.

Supplementary Figure 5 Box plots of the gene expression of GSE171110 dataset before **(A)** and after **(B)** normalization.

Supplementary Figure 6 Box plots of the gene expression of GSE152418 dataset before **(A)** and after **(B)** normalization.

Supplementary Table 1 The expression of COVID-19 differentially expressed genes.

Supplementary Table 2 The expression of HIV differentially expressed genes.

Supplementary Table 3 Volcano plots of COVID-19 differentially expressed genes.

Supplementary Table 4 Volcano plots of HIV differentially expressed genes.

Supplementary Table 5 Common DEGs between COVID-19 and HIV.

Supplementary Table 6 The TFs and their interactions with DEGs.

Supplementary Table 7 The microRNAs and their interactions with DEGs.

Supplementary Table 8 The potential drugs for the treatment of COVID-19 and HIV.

Supplementary Table 9 Regulatory interactions between common DEGs and diseases.

## Abbreviations

COVID-19, severe coronavirus disease 2019; SARS-CoV-2, severe acute respiratory syndrome coronavirus 2; HIV, human immunodeficiency virus; GEO, Gene Expression Omnibus; DEG, differentially expressed gene; PPI, protein-protein interaction; TF, transcription factor; miRNAs, microRNAs; ARDS, acute respiratory distress syndrome; AIDS, Acquired immunodeficiency syndrome; GO, Gene Ontology; KEGG, Kyoto Encyclopedia of Genes and Genomes; BP, biological process; CC, cell composition; MF, molecular function; DSigDB, Drug Signatures Database; AUC, area under curve.
