# Supplementary figures and images for: Blood transcriptome analysis revealed the crosstalk between COVID-19 and HIV
Source: Front Immunol. 2022 Oct 28;13:1008653. doi: 10.3389/fimmu.2022.1008653 (PMC9650272; doi:10.3389/fimmu.2022.1008653)

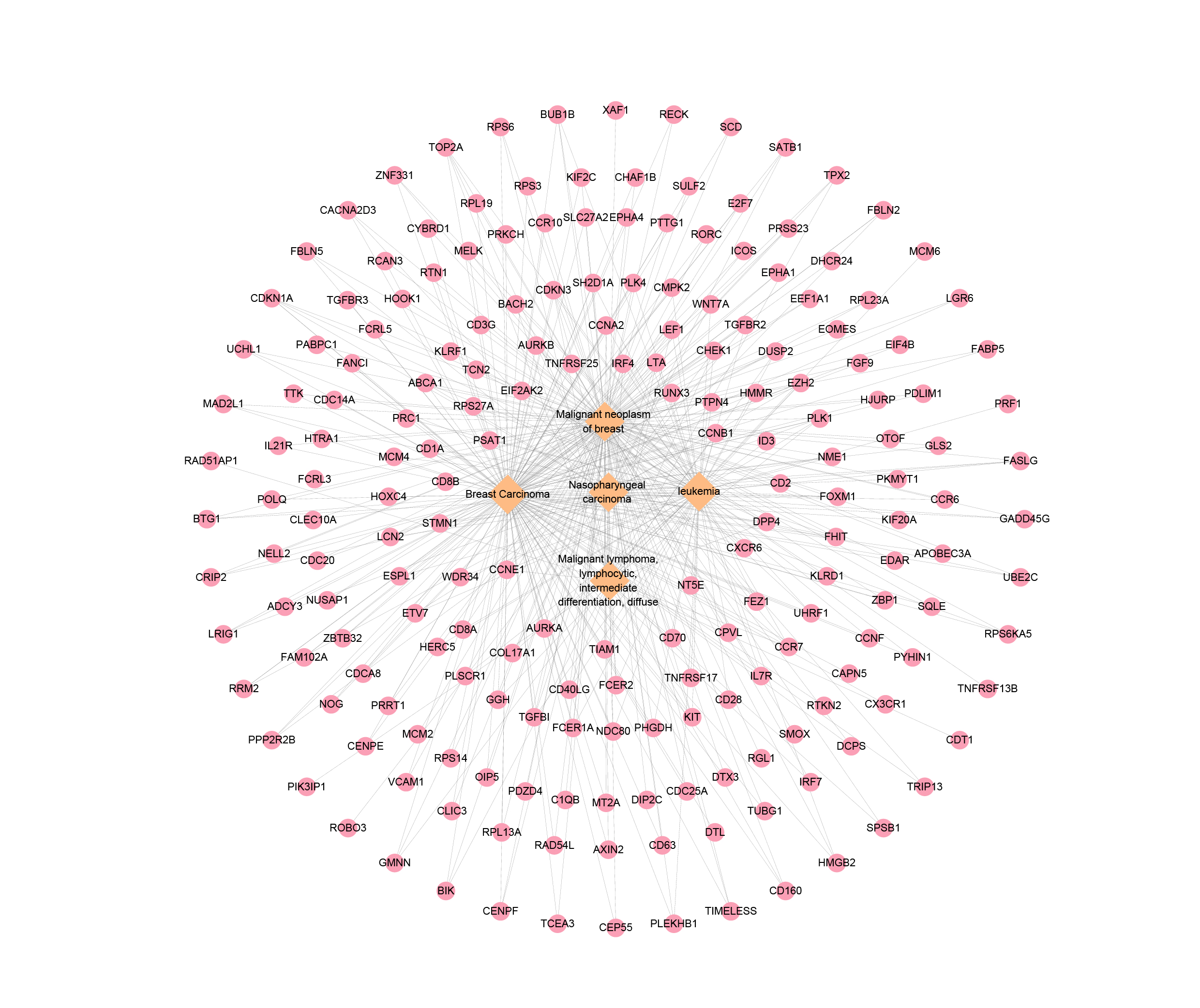

Supplement: Supplementary Figure 1 — Regulatory interactions between common DEGs and diseases. [file Image_1.jpeg]

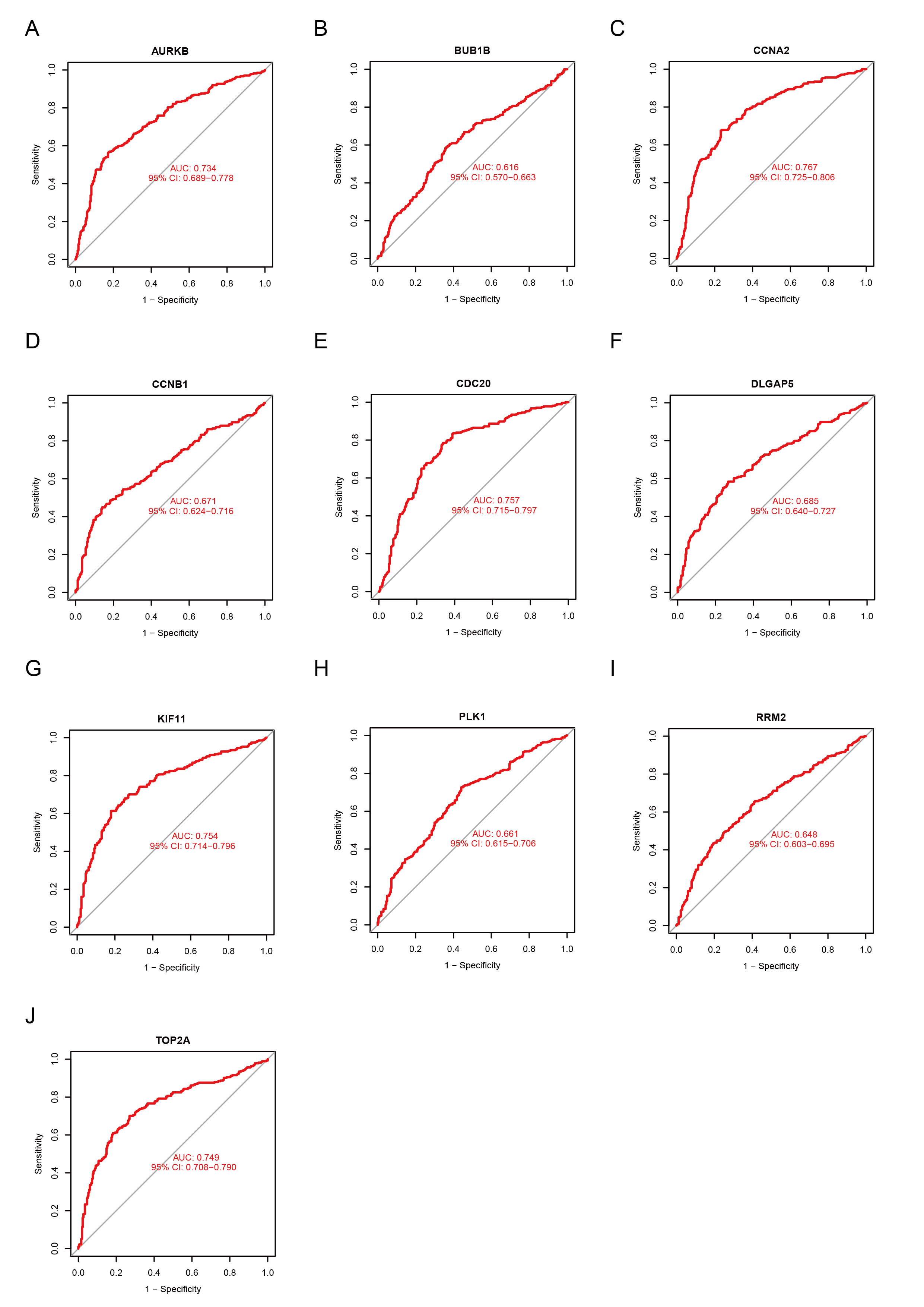

Supplement: Supplementary Figure 2 — ROC analysis of the hub DEGs in the HIV dataset. [file Image_2.jpeg]

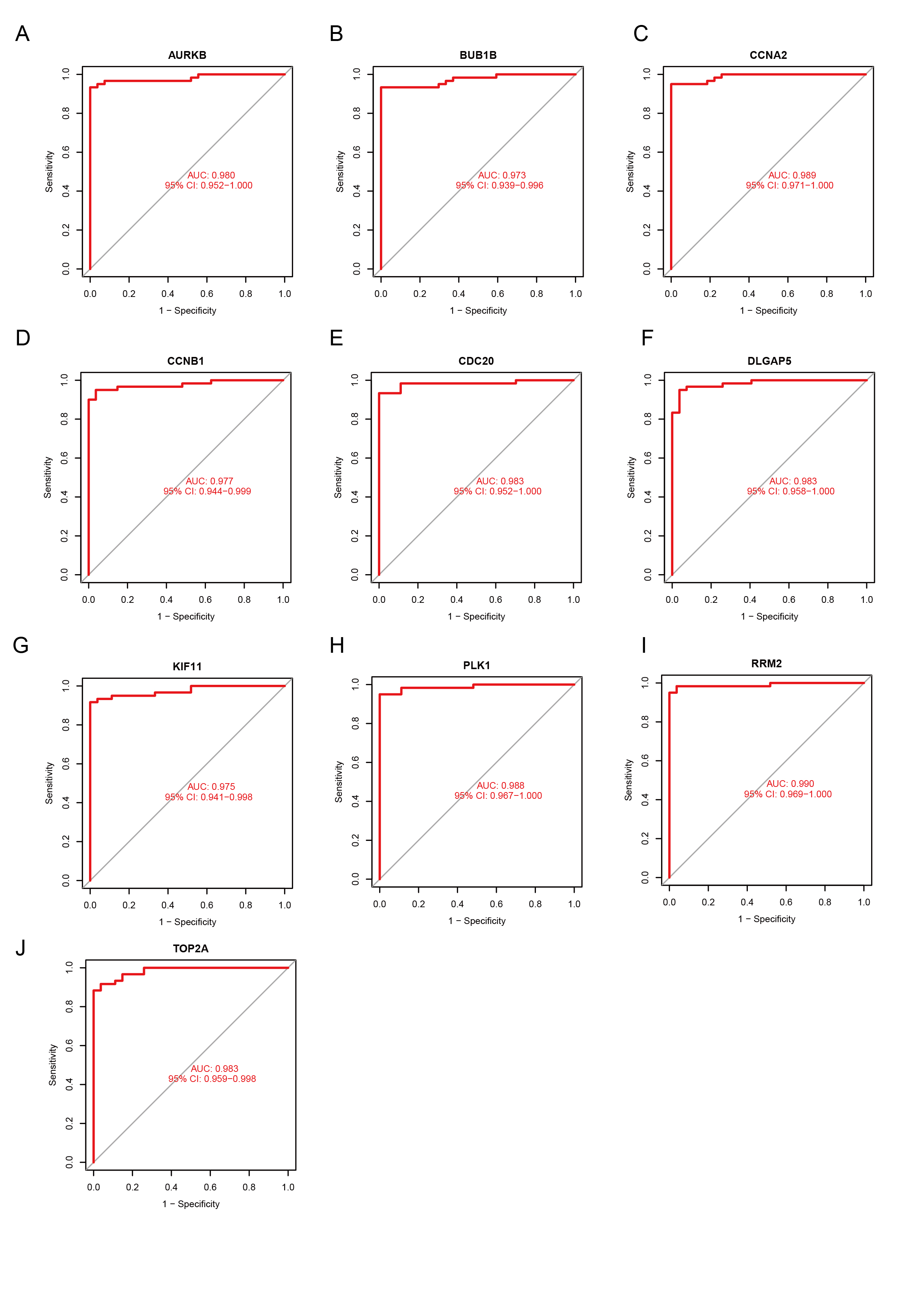

Supplement: Supplementary Figure 3 — ROC analysis of the hub DEGs in the COVID-19 dataset. [file Image_3.jpeg]

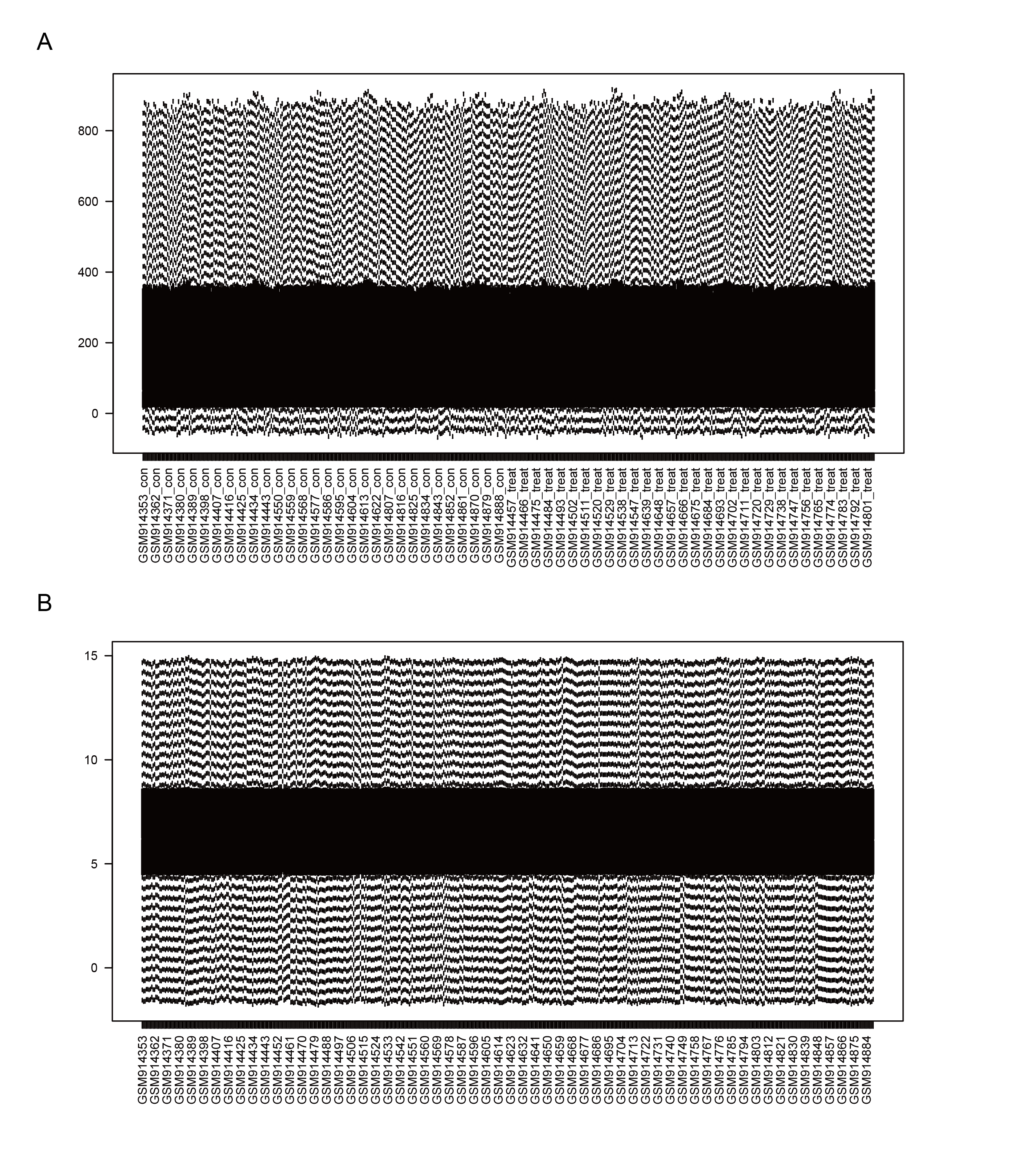

Supplement: Supplementary Figure 4 — Box plots of the gene expression of GSE37250 dataset before (A) and after (B) normalization. [file Image_4.jpeg]

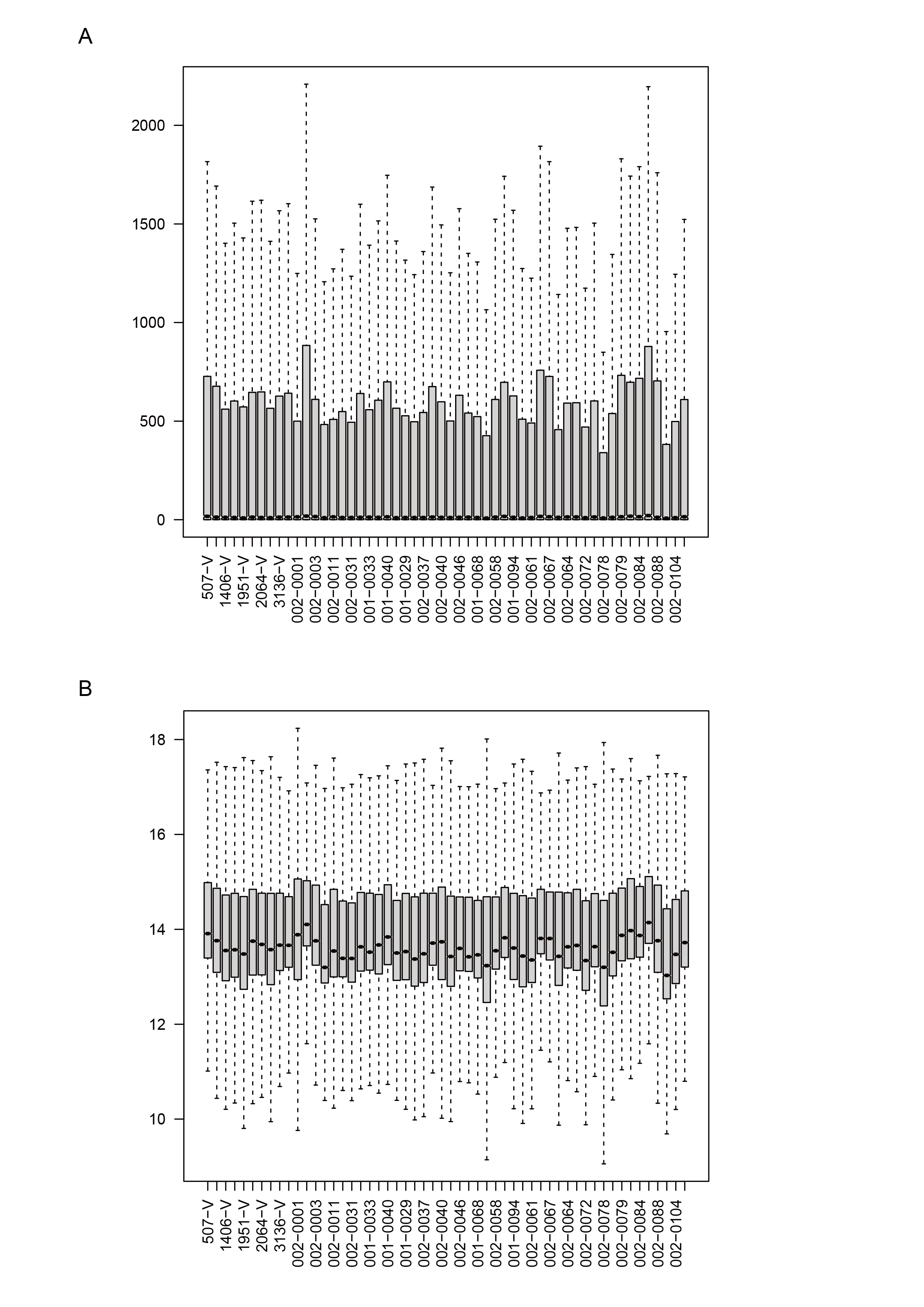

Supplement: Supplementary Figure 5 — Box plots of the gene expression of GSE171110 dataset before (A) and after (B) normalization. [file Image_5.jpeg]

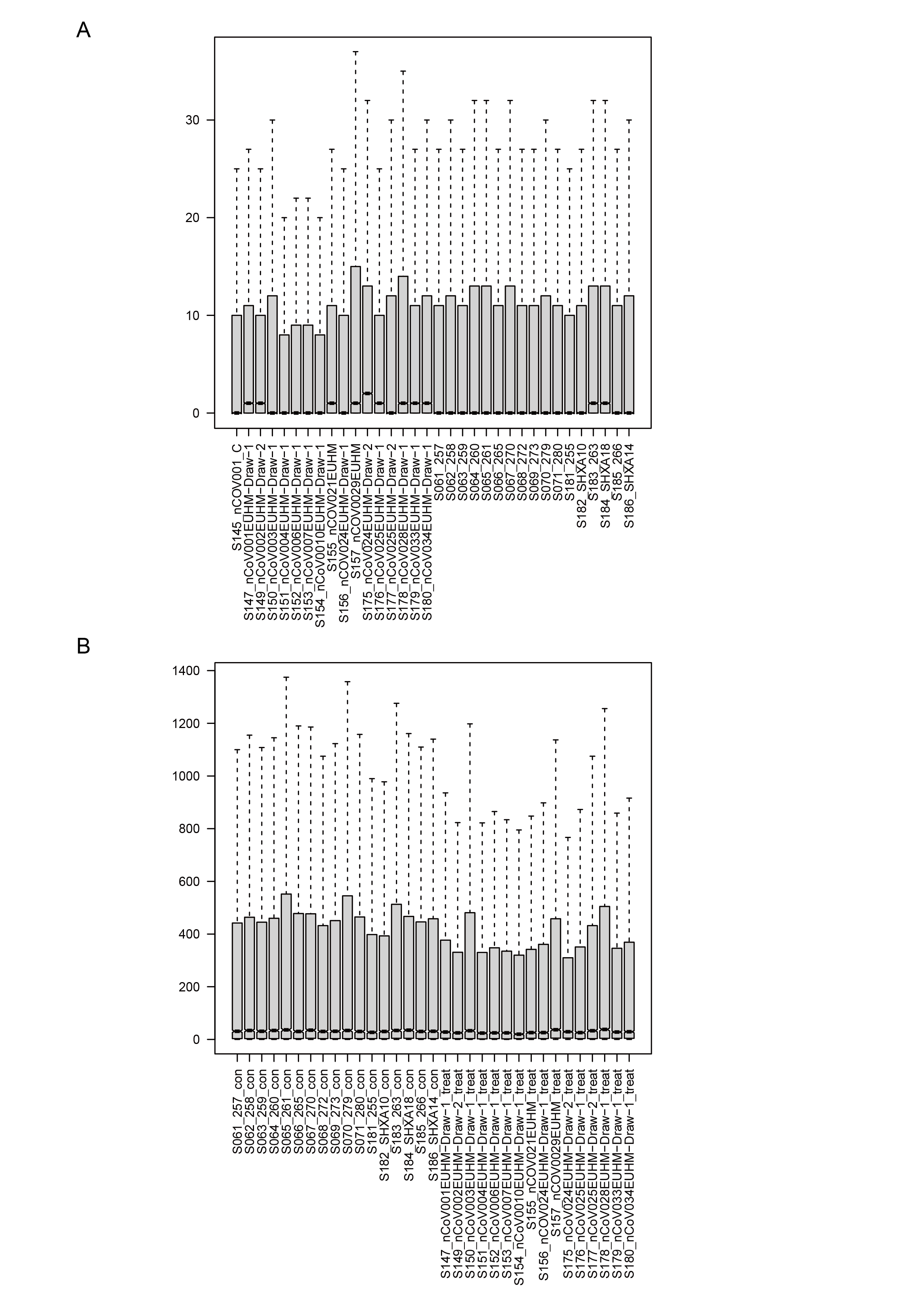

Supplement: Supplementary Figure 6 — Box plots of the gene expression of GSE152418 dataset before (A) and after (B) normalization. [file Image_6.jpeg]
